# Author Correction: Epithelial TGFβ engages growth-factor signalling to circumvent apoptosis and drive intestinal tumourigenesis with aggressive features

**DOI:** 10.1038/s41467-023-36266-w

**Published:** 2023-01-31

**Authors:** Dustin J. Flanagan, Raheleh Amirkhah, David F. Vincent, Nuray Gunduz, Pauline Gentaz, Patrizia Cammareri, Aoife J. McCooey, Amy M. B. McCorry, Natalie C. Fisher, Hayley L. Davis, Rachel A. Ridgway, Jeroen Lohuis, Joshua D. G. Leach, Rene Jackstadt, Kathryn Gilroy, Elisa Mariella, Colin Nixon, William Clark, Ann Hedley, Elke K. Markert, Douglas Strathdee, Laurent Bartholin, Keara L. Redmond, Emma M. Kerr, Daniel B. Longley, Fiona Ginty, Sanghee Cho, Helen G. Coleman, Maurice B. Loughrey, Alberto Bardelli, Timothy S. Maughan, Andrew D. Campbell, Mark Lawler, Simon J. Leedham, Simon T. Barry, Gareth J. Inman, Jacco van Rheenen, Philip D. Dunne, Owen J. Sansom

**Affiliations:** 1 Cancer Research UK Beatson Institute, Glasgow, UK; 2 Department of Biochemistry and Molecular Biology, Monash University, Melbourne, Australia; 3 Cancer Program, Biomedicine Discovery Institute, Monash University, Melbourne, Australia; 4 The Patrick G. Johnston Centre for Cancer Research, Queen’s University Belfast, Belfast, UK; 5 Wellcome Trust Centre for Human Genetics, University of Oxford, Oxford, UK; 6 Department of Molecular Pathology, Oncode Institute, The Netherlands Cancer Institute, Amsterdam, The Netherlands; 7 Institute of Cancer Sciences, University of Glasgow, Glasgow, UK; 8 Heidelberg Institute for Stem Cell Technology and Experimental Medicine (HI-STEM gGmbH) and Deutsches Krebsforschungszentrum (DKFZ), Heidelberg, Germany; 9 Department of Oncology, University of Torino, Candiolo Torino, Italy; 10 University of Newcastle upon Tyne, Newcastle, UK; 11 INSERM Centre de Recherche en Cancérologie de Lyon, Lyon, France; 12 GE Global Research Center, Niskayuna, NY USA; 13 Centre for Public Health, Queen’s University Belfast, Belfast, UK; 14 Department of Cellular Pathology, Belfast Health and Social Care Trust, Belfast, UK; 15 CRUK/MRC Oxford Institute for Radiation Oncology, University of Oxford, Oxford, UK; 16 Bioscience, Oncology R&D, AstraZeneca, Cambridge, UK

**Keywords:** Cancer models, Targeted therapies, Colon cancer

Correction to: *Nature Communications* 10.1038/s41467-022-35134-3, published online 07 December 2022

In this article the author name Nuray Gunduz was incorrectly written as Nuray Gundaz. The original article has been corrected.

