# Supplementary material for: Epithelial TGFβ engages growth-factor signalling to circumvent apoptosis and drive intestinal tumourigenesis with aggressive features
Source: Nat Commun. 2022 Dec 7;13:7551. doi: 10.1038/s41467-022-35134-3 (PMC9729215; doi:10.1038/s41467-022-35134-3)
Supplement: Supplementary file 1 — Supplementary Information [file 41467_2022_35134_MOESM1_ESM.pdf]

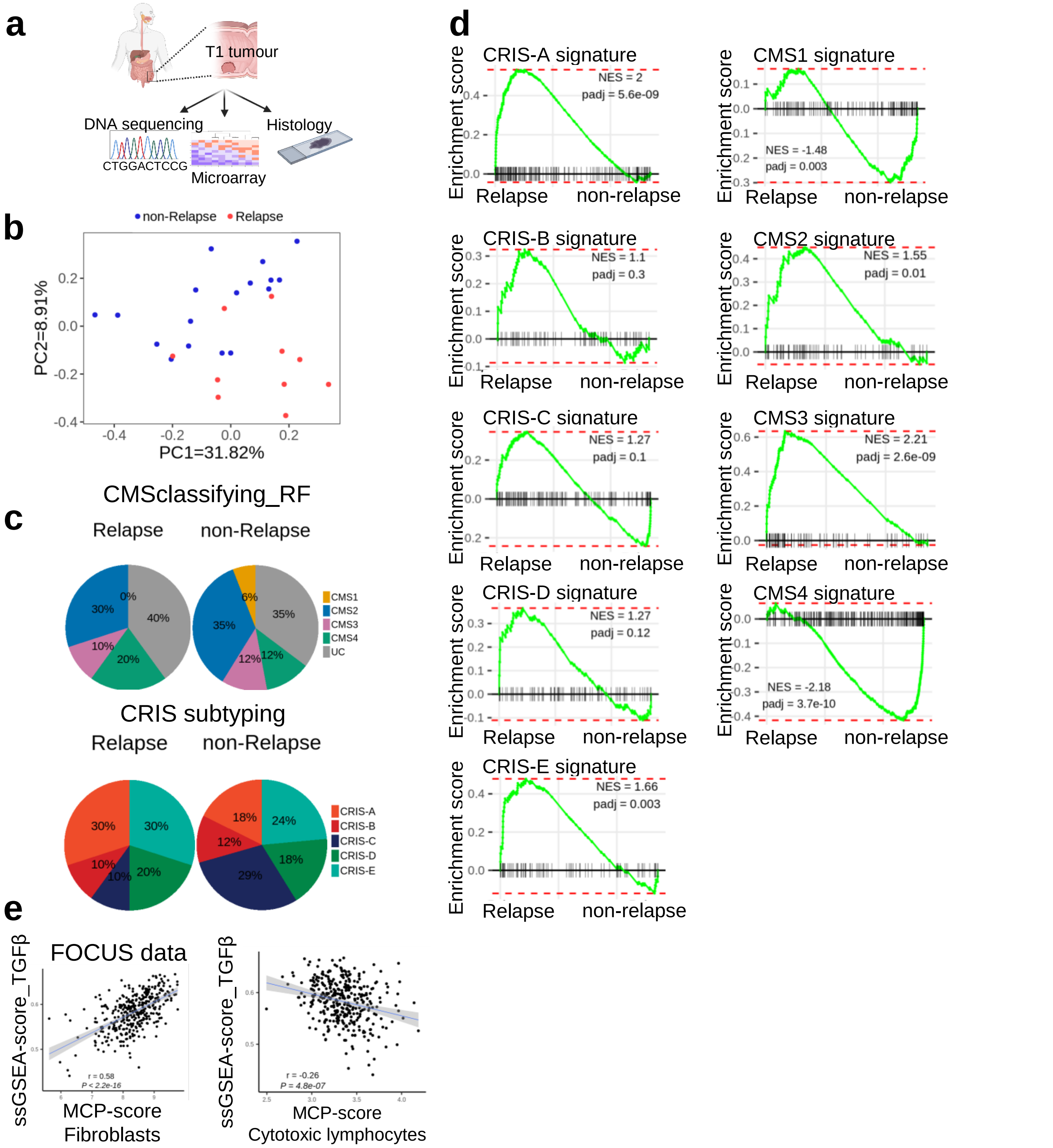

Supplementary Figure 1. CMS and CRIS classifiers to identify pT1 relapse cases.
a, Schematic of sample collection and downstream analysis of pT1 patients. **b**, Principal component analysis (PCA) plot depicting the clustering of transcriptomes from relapse (n=10; red) and non-relapse (n=17; blue) pT1 cohort primary tumours. **c**, Pie charts showing CMS (top) and CRIS (bottom) classification of relapse (n=17, left) and non-relapse (n=10, right) pT1 tumours. Percentages indicate the prevalence of CMS and CRIS subclasses among pT1 tumours. **d**, GSEA of CRIS and CMS signatures in relapse vs non-relapse cases using fgsea. CRIS subtype padj= 5.6e-09 (A); 0.3 (B); 0.1 (C); 0.12 (D) and 0.003 (E) respectively. CMS subtype padj=0.003 (1); 0.01 (2); 2.6e-09 (3); and 3.7e-10 (4). NES=Normalized Enrichment Score; padj, adjusted p-value (computed and corrected for multiple testing using the Benjamini–Hochberg procedure). **e**, Correlation analysis between ssGSEA Hallmark TGFβ signalling gene set scores and the transcriptome-based MCP-counter scores for fibroblast (left) and cytotoxic T lymphocytes (right) in the FOCUS metastatic CRC clinical trial cohort. Left plot, two-sided P=<2.2e-16, r=0.58, right plot, two-sided P=4.8e-07, r= -0.26. r = Pearson correlation coefficient. Grey region shows the 95% CI for predictions from a lm.

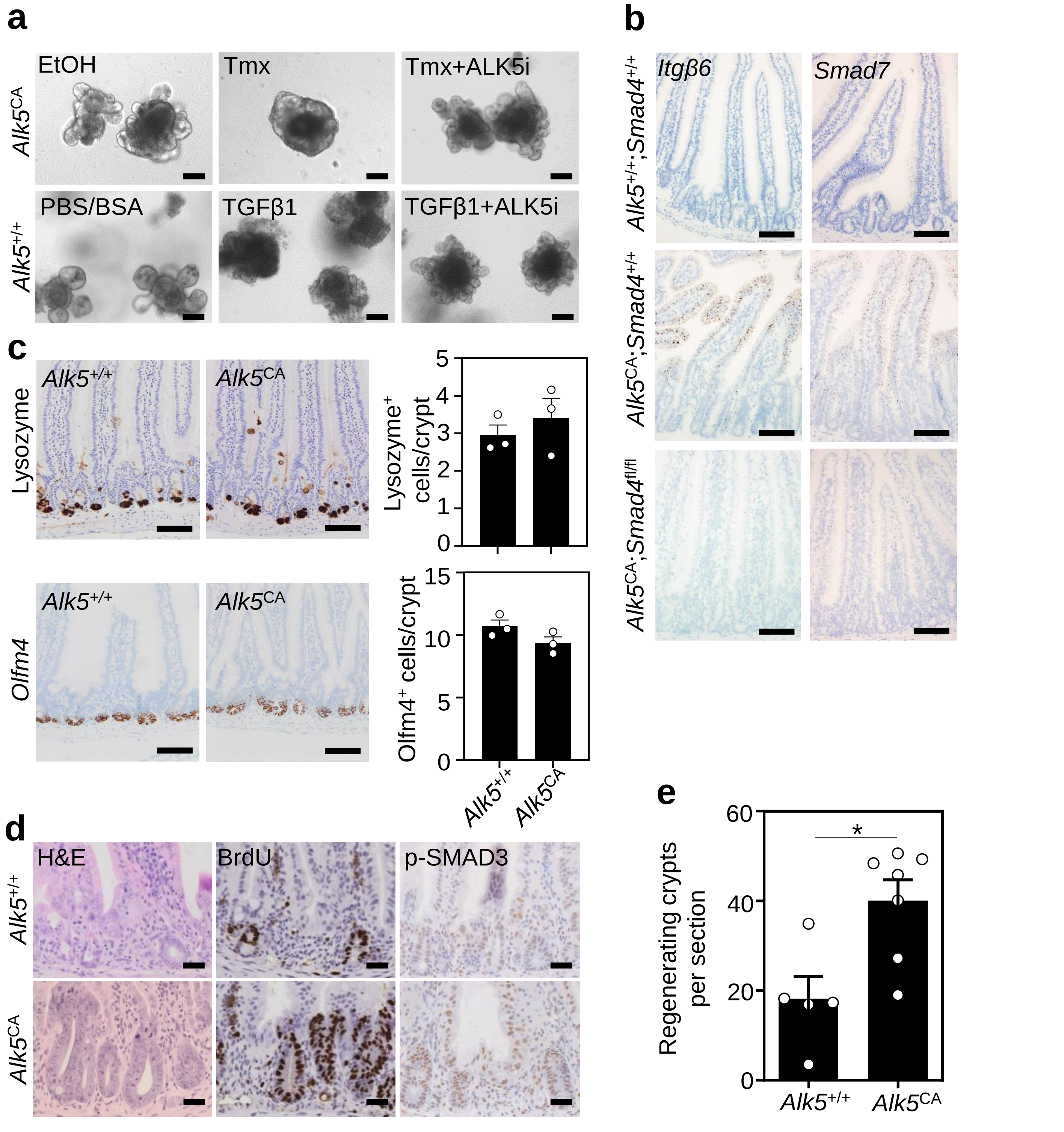

Supplementary Figure 2. Epithelial cell-intrinsic TGFβ signalling promotes intestinal regeneration.
a, Representative images of small intestinal organoids derived from *VilCre*^{ER}*;Alk5*^{CA} (*Alk5*^{CA}) and *VilCre*^{ER}*;Alk5*$^{+/+}$ (*Alk5*$^{+/+}$) mice 5 days following 5 days of *in vitro* treatment with vehicle control (EtOH for tamoxifen; PBS/BSA for TGFβ1), tamoxifen (Tmx), TGFβ1, and either Tmx or TGFβ1 in combination with ALK5i. n=3 organoid lines per group. Treatments were repeated twice. Scale bar, 50 µm. **b**, Representative ISH for *Itgβ6* and *Smad7* of *VilCre*^{ER}*;Alk5*$^{+/+}$*;Smad4*$^{+/+}$ (*Alk5*$^{+/+}$*;Smad4*$^{+/+}$) *VilCre*^{ER}*;Alk5*^{CA}*;Smad4*$^{+/+}$ (*Alk5*^{CA}*;Smad4*$^{+/+}$) and *VilCre*^{ER}*;Alk5*^{CA}*;Smad4*$^{fl/fl}$ (*Alk5*^{CA}*;Smad4*$^{fl/fl}$) mice 4 days post-tamoxifen induction. Scale bar, 100 µm. Staining was performed on tissue sections from n=4 mice per genotype. **c**, Left, representative lysozyme and Olfm4 staining of *VilCre*^{ER}*;Alk5*$^{+/+}$ (*Alk5*$^{+/+}$) and *VilCre*^{ER}*;Alk5*^{CA} (*Alk5*^{CA}) mice 60 days post-tamoxifen induction. Scale bar, 100 µm. Right, quantification of lysozyme^{+} and Olfm4^{+} cells. n=3 mice of each genotype. Data are ± s.e.m; one-tail Mann–Whitney U-test. **d**, Representative H&E and IHC for BrdU and p-SMAD3 in tamoxifen-induced *VilCre*^{ER}*;Alk5*$^{+/+}$ (*Alk5*$^{+/+}$) and *VilCre*^{ER}*;Alk5*^{CA} (*Alk5*^{CA}) mice 72hrs following 10Gy whole body γ-radiation. Scale bar, 100 µm. **e**, Quantification of regenerating crypts per small intestinal cross-section from mice described in **c**. n=5 *Alk5*$^{+/+}$, n=7 *Alk5*^{CA} mice. Data are ± s.e.m: *P=0.01. Two-tail Mann–Whitney U-test.

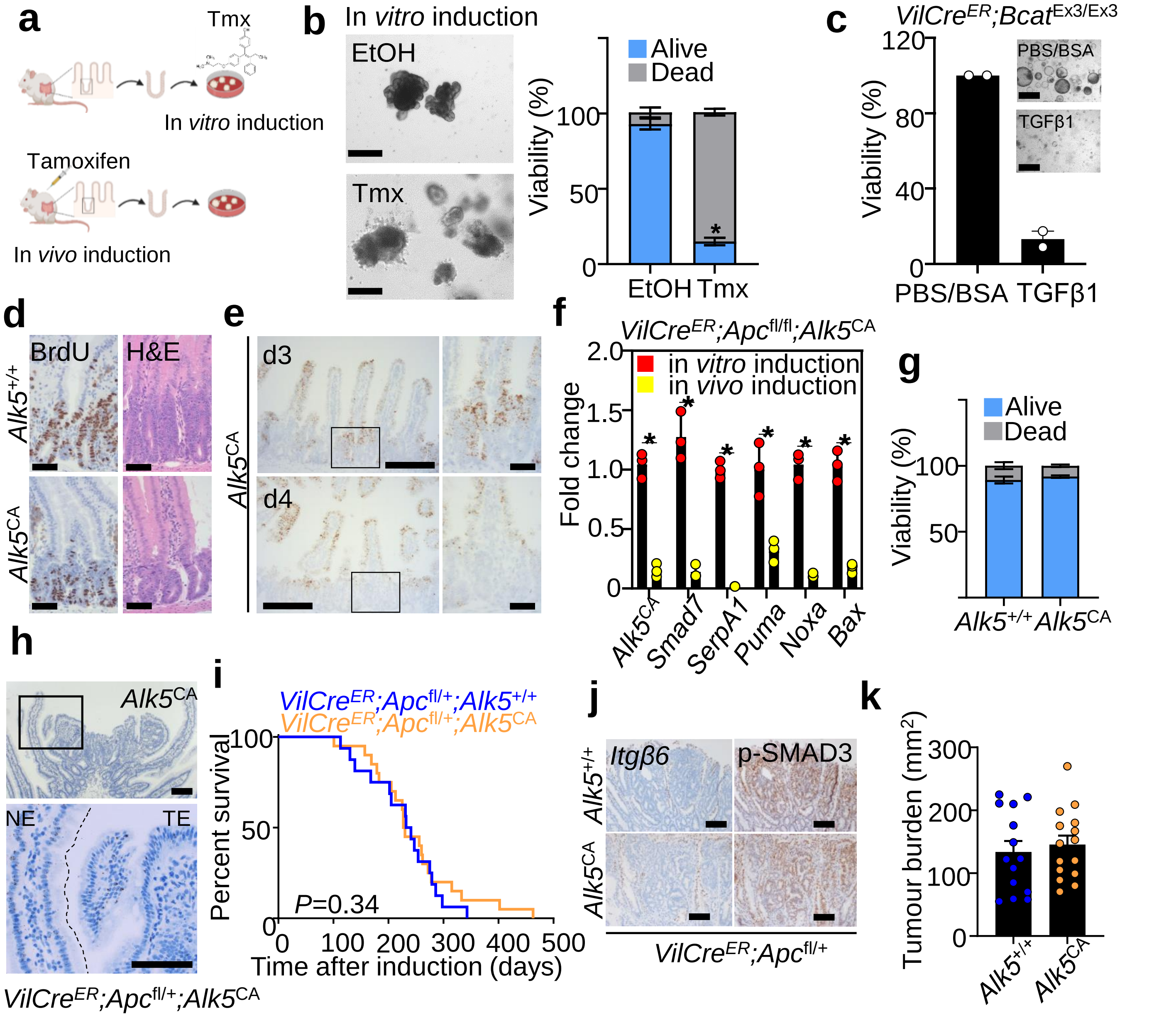

Supplementary Figure 3. TGFβ induces potent cytostatic and cytotoxic effects in *Apc*-mutant organoids.
a, Schematic illustrating the derivation of murine intestinal organoids and gene recombination strategy; *in vitro* (top) and *in vivo* (bottom). **b**, Left, representative images of *VilCre*^{ER};*Apc*$^{fl/fl}$;*Alk5*^{CA} organoids following 5 days of treatment with vehicle (EtOH) or 4-hydroxytamoxifen (Tmx) – *in vitro* induction. Right, relative organoid viability (dead; grey vs alive; blue) 5 days post treatment. n=3 individual organoid lines per group, each in technical triplicate. Data are ± s.e.m; *P=0.05, one-tail Mann–Whitney U-test. Scale bar, 100 μm. **c**, Relative viability of *in vivo* induced *VilCre*^{ER};*Bcat*$^{Ex3/Ex3}$ small intestinal organoids 4 days post-treatment with vehicle (PBS/BSA) or TGFβ1 (5 ng/ml). Viability measured 4 days post-treatment. n=2 individual organoid lines per group, each in technical triplicate. Data are ± s.e.m. Inset, representative images of organoids described above 4 days after treatment. Scale bar, 200 μm. **d**, Representative BrdU and H&E staining of *VilCre*^{ER};*Apc*$^{fl/fl}$;*Alk5*$^{+/+}$ (*Alk5*$^{+/+}$) and *VilCre*^{ER};*Apc*$^{fl/fl}$;*Alk5*^{CA} (*Alk5*^{CA}) mice 3 days post-tamoxifen. Staining was performed on tissue sections from n=3 mice per genotype. Scale bar, 100 μm. **e**, Representative *Alk5*^{CA} ISH staining of small intestinal tissue from *VilCre*^{ER};*Apc*$^{fl/fl}$;*Alk5*^{CA} mice 3 (d3) and 4 (d4) days post-tamoxifen. Boxed areas show close-up of epithelial *Alk5*^{CA} expression. Staining was performed on tissue sections from n=3 mice per genotype. Scale bar, 100 μm. **f**, qPCR for TGFβ-target and apoptosis genes expressed by *VilCre*^{ER};*Apc*$^{fl/fl}$;*Alk5*^{CA} organoids induced *in vitro* (red) and *in vivo* (yellow). n=3 individual organoid lines per group, each in technical triplicate. Data are ± s.e.m; *P=0.05, one-tail Mann–Whitney U-test. **g**, Relative organoid viability of small intestinal organoids derived from *VilCre*^{ER};*Apc*$^{fl/fl}$;*Alk5*$^{+/+}$ (*Alk5*$^{+/+}$) and *VilCre*^{ER};*Apc*$^{fl/fl}$;*Alk5*^{CA} (*Alk5*^{CA}) mice 4 days post-tamoxifen – *in vivo* induction. n=3 individual organoid lines per group, each in technical triplicate. Data are ± s.e.m; *P=0.05, one-tail Mann–Whitney U-test. **h**, Representative *Alk5*^{CA} ISH on tumour tissue from *VilCre*^{ER};*Apc*$^{fl/+}$;*Alk5*^{CA} mice. Boxed area is magnification of lower panel, with dashed line denoting the boundary between tumour epithelium (TE) and the adjacent normal epithelium (NE). Scale bar, 100 μm. **i**, Survival plot for *VilCre*^{ER};*Apc*$^{fl/+}$;*Alk5*$^{+/+}$ (n=16, blue) and *VilCre*^{ER};*Apc*$^{fl/+}$;*Alk5*^{CA} (n=20, orange) mice aged until clinical endpoint following tamoxifen induction. P=0.34, log-rank test. **j**, Representative *Itgβ6* (ISH) and p-SMAD3 (IHC) staining on tumour tissue from mice described in **i**. Scale bar, 200 μm. **k**, Total intestinal tumour burden per mouse (area) from *VilCre*^{ER};*Apc*$^{fl/+}$;*Alk5*$^{+/+}$ (n=14, blue) and *VilCre*^{ER};*Apc*$^{fl/+}$;*Alk5*^{CA} (n=15, orange) mice aged until clinical endpoint following tamoxifen induction. Data are ± s.e.m; P=0.71. Two-tail Mann–Whitney U-test.

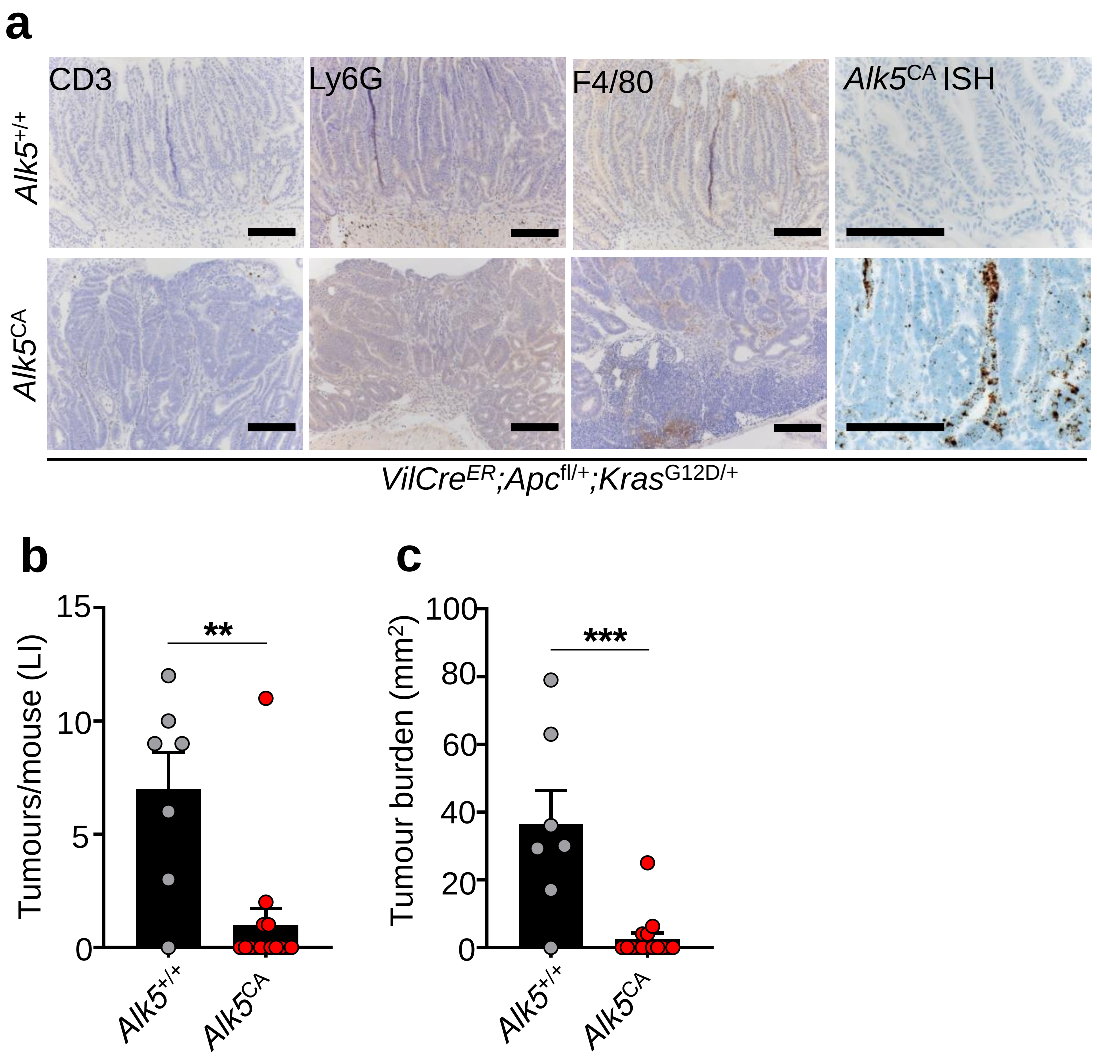

Supplementary Figure 4. *Apc.Kras-mutant cells tolerate constitutive ALK5 signalling.*
a, Representative IHC staining for T cells (CD3), neutrophils (Ly6G) and macrophages (F4/80) on tumour tissue from *VilCre^{ER};Apc$^{fl/+}$;Kras$^{G12D/+}$;Alk5$^{+/+}$* (*Alk5$^{+/+}$*) and *VilCre^{ER};Apc$^{fl/+}$;Kras$^{G12D/+}$;Alk5^{CA}* (*Alk5^{CA}*) mice. Right, *Alk5^{CA}* ISH on tumour tissue. Note, *Alk5^{CA}* mice maintain expression of *Alk5^{CA}* in the presence of concurrent *Apc* and *Kras* mutations. Scale bar, 100 μm. **b-c**, Large intestinal (LI) tumour number (**b**) and burden (**c**) per mouse from *VilCre^{ER};Apc$^{fl/+}$;Kras$^{G12D/+}$;Alk5$^{+/+}$* (n=7, grey) and *VilCre^{ER};Apc$^{fl/+}$;Kras$^{G12D/+}$;Alk5^{CA}* (n=15, red) mice. Data are ± s.e.m; **P=0.002 (**b**), ***P=5.0x10^{-3} (**c**). Two-tail Mann–Whitney U-test for **b** and **c**.

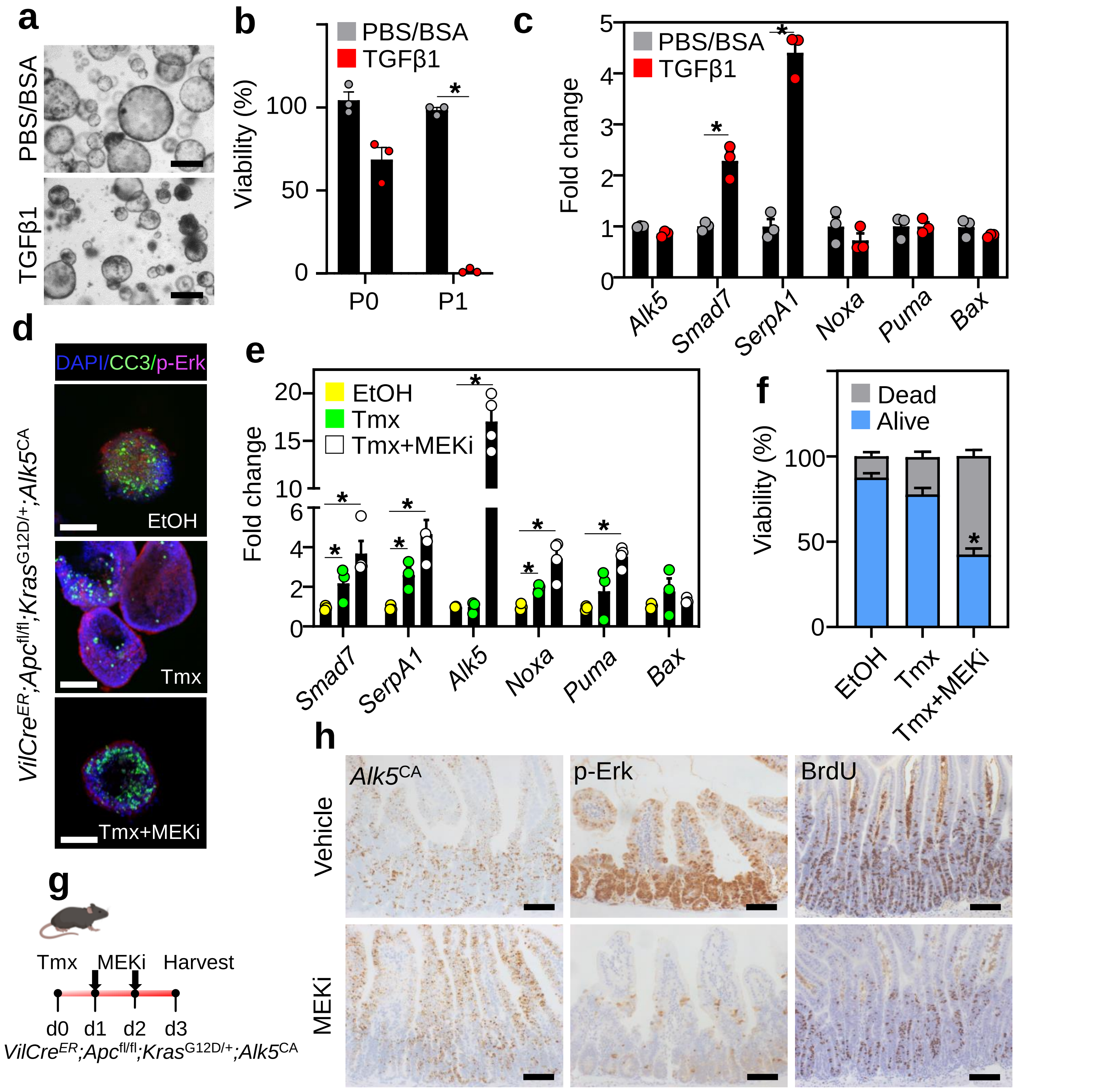

Supplementary Figure 5. Mutant *Kras* buffers against epithelial TGFβ signalling.
a, Representative images of small intestinal organoids derived from tamoxifen-induced *VilCre*^{ER}*;Apc*$^{fl/fl}$*;Kras*$^{G12D/+}$ mice 5 days post vehicle (PBS/BSA, grey) or TGFβ1 (red) treatment. Scale bar, 100 μm. **b**, Relative cell viability of organoids described in **a** before (P0) or 4 days following passage (P1). n=3 individual organoid lines per group. Data are ± s.e.m; *P=0.05. One-tail Mann–Whitney U-test. **c**, qPCR for TGFβ-target and apoptotic genes expressed by *VilCre*^{ER}*;Apc*$^{fl/fl}$*;Kras*$^{G12D/+}$ organoids 5 days following PBS/BSA or TGFβ1 treatment. n=3 individual organoid lines per group, each in technical triplicate. Data are ± s.e.m; *P=0.05. One-tail Mann–Whitney U-test. **d**, Representative immunofluorescent staining for cleaved caspase-3 (CC3) and p-ERK in *VilCre*^{ER}*;Apc*$^{fl/fl}$*;Kras*$^{G12D/+}$*;Alk5*^{CA} organoids following treatment with vehicle control (EtOH) or 4-hydroxytamoxifen (Tmx) ± MEK 1/2 inhibitor (MEKi). Staining was performed on n=3 independent organoid lines per treatment group. Scale bar, 50 μm. **e**, qPCR for TGFβ-target and apoptotic genes expressed by organoids described in **d**. n=3 individual organoid lines per group; EtOH (yellow), Tmx (green), Tmx+MEKi (white) except n=4 Tmx+MEKi, each in technical triplicate. Data are ± s.e.m; *P=0.05 (Tmx), *P=0.02 (Tmx+MEKi). One-tail Mann–Whitney U-test. **f**, Relative viability of organoids described in **d**. n=3 biological replicates per group; dead (grey), alive (blue), each in technical triplicate. Data are ± s.e.m; *P=0.05. One-tail Mann–Whitney U-test. **g**, Timeline schematic illustrating tamoxifen induction, treatment regimen and tissue harvesting of *VilCre*^{ER}*;Apc*$^{fl/fl}$*;Kras*$^{G12D/+}$*;Alk5*^{CA} mice. **h**, Representative *Alk5*^{CA} ISH and p-ERK and BrdU IHC of small intestinal tissue from *VilCre*^{ER}*;Apc*$^{fl/fl}$*;Kras*$^{G12D/+}$*;Alk5*^{CA} mice 3 days post-tamoxifen injection and subsequent treatment with vehicle (HPMC) or MEKi. Staining was performed on tissue sections from n=3 mice per treatment group. Scale bar, 100 μm.

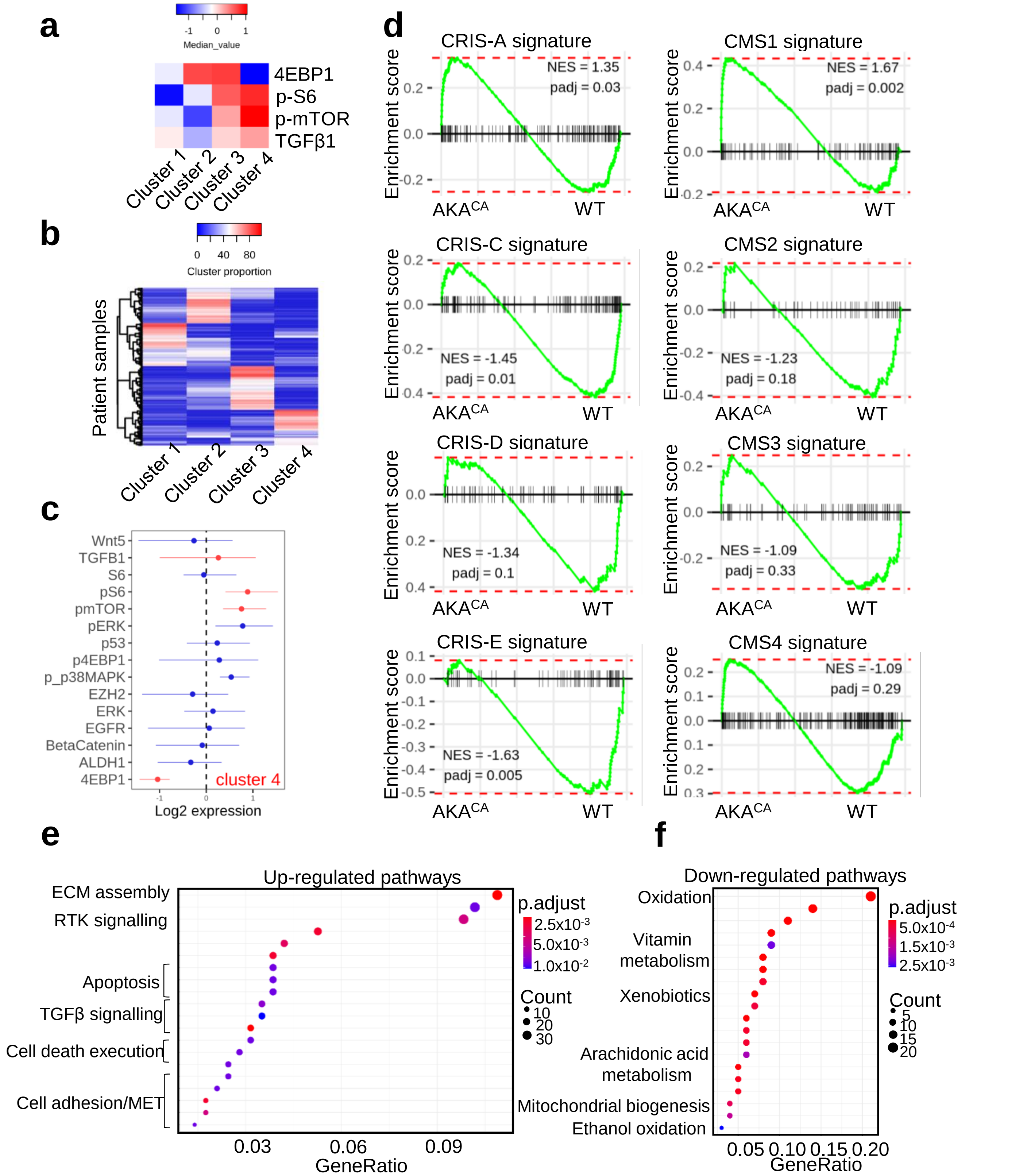

Supplementary Figure 6. Molecular clustering of patient T1 tumours and *Apc.Kras.Alk5Ca* mutant tissue.
a, Heatmap of 4EBP1, p-mTOR, p-S6 and TGFβ1 staining in 5.6×10^5 tumour cells from a cohort of 282 stage II CRC patients. The median marker intensities for each of these markers at single-cell level were used to visualize the association of each cluster of stage II CRC patients with each marker. **b**, Heat map of cluster enrichment in 282 stage II CRC patients based on the proportion of each cluster across patients. **c**, Lollipop plot showing the expression profile of key components of the mTOR and TGFβ pathways in cluster 4. “Lollipop” lines originate at the average expression of proteins in all cells of cluster 4 (n=107060). Lines to the left and right of the middle point (mean expression) show lower and upper quantile value. **d**, CRIS and CMS signature enrichment performed on the shrunken log expression ratio of *VilCre*^{ER};*Apc*$^{fl/fl}$;*Kras*$^{G12D/+}$;*Alk5*^{CA} (AKA^{CA}) vs wild type (WT) intestinal tissue 3 days post tamoxifen injection using fgsea. NES=Normalized Enrichment Score; padj, adjusted p-value (computed and corrected for multiple testing using the Benjamini–Hochberg procedure). **e-f**, Reactome pathway analysis showing up-regulated (**e**) and down-regulated (**f**) pathways in intestinal tissues from *VilCre*^{ER};*Apc*$^{fl/fl}$;*Kras*$^{G12D/+}$;*Alk5*^{CA} mice compared to *VilCre*^{ER};*Apc*$^{fl/fl}$;*Kras*$^{G12D/+}$;*Alk5*$^{+/+}$ mice 3 days post-tamoxifen. n=3 mice per group.

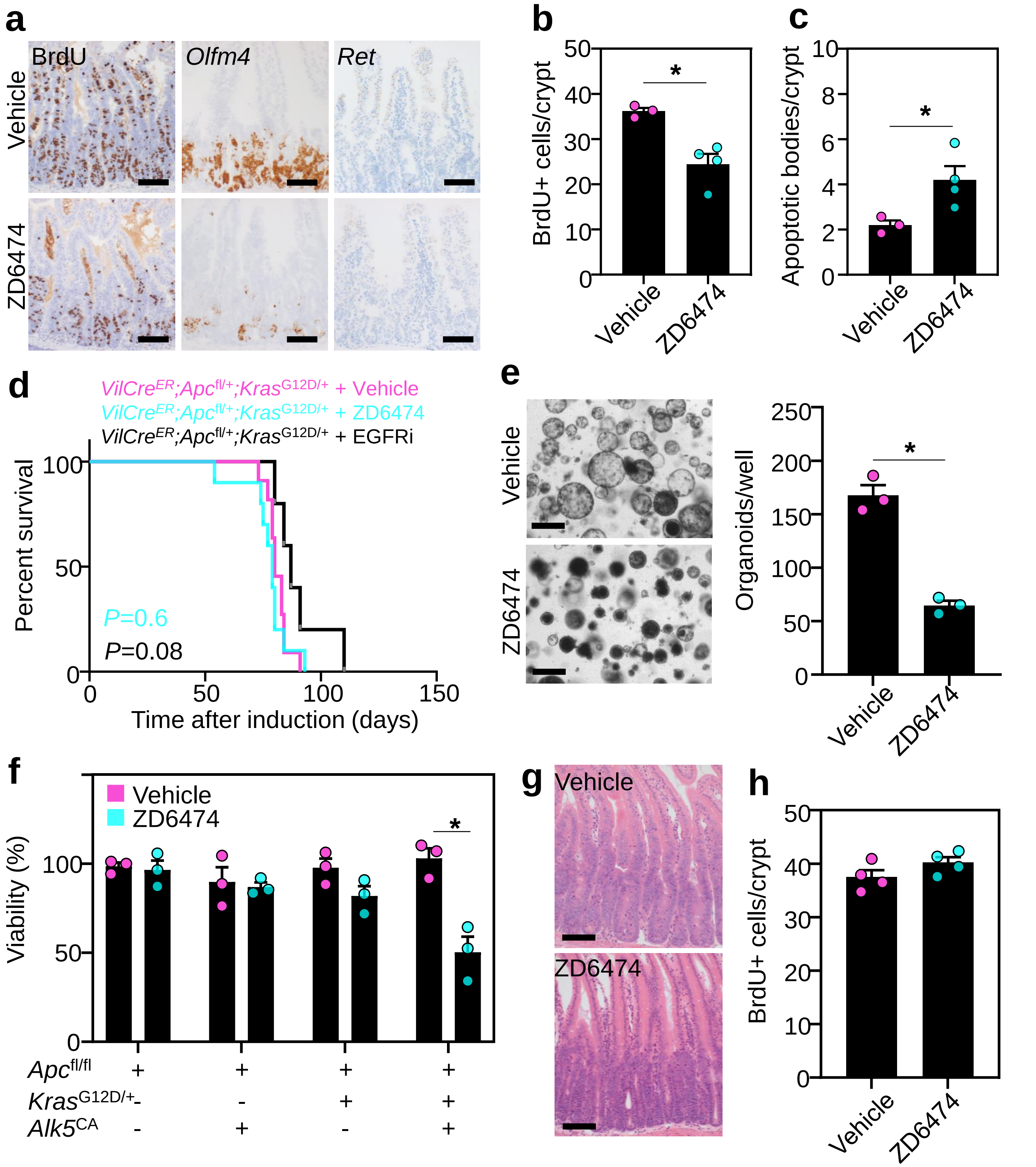

Supplementary Figure 7. Epithelial TGFβ sensitise cells to inhibitors of growth-factor signalling.
a, Representative IHC for BrdU, and ISH for *Olfm4* and *Ret* on small intestinal tissue from *VilCre^{ER};Apc$^{fl/fl}$;Kras$^{G12D/+}$;Alk5^{CA}* mice 3 days post-tamoxifen following daily treatment with vehicle control or ZD6474. Scale bar, 100 μm. **b-c**, Quantification of BrdU-positive (**b**) and apoptotic cells (**c**) in mice described in **a**. n=3 vehicle (pink), n=4 ZD6474 (aqua). Data are ± s.e.m; *P=0.05. One-tail Mann–Whitney U-test for **b** and **c**. **d**, Survival plot for *VilCre^{ER};Apc$^{fl/+}$;Kras$^{G12D/+}$* mice treated daily with vehicle, ZD6474 or EGFR inhibitor (EGFRi) and aged until clinical endpoint following tamoxifen induction. n=10 vehicle(pink), n=11 ZD6474 (aqua), n=5 EGFRi (black) mice. P=0.6 (ZD6474), P=0.08 (EGFRi); log-rank test. **e**, Representative images (left) and quantification (right) of small intestinal organoids derived from tamoxifen-induced *VilCre^{ER};Apc$^{fl/fl}$;Kras$^{G12D/+}$;Alk5^{CA}* mice 5 days following supplementation with vehicle (DMSO, pink) or ZD6474 (aqua). n=3 biological replicates per group, each in technical triplicate. Data are ± s.e.m; *P=0.05. One-tail Mann–Whitney U-test. Scale bar, 100 μm. **f**, Relative viability of small intestinal organoids of the indicated genotypes 5 days following treatment with vehicle (DMSO, pink) or ZD6474 (aqua). n=3 independent organoid lines per group, each in technical triplicate. Data are ± s.e.m; *P=0.05. One-tail Mann–Whitney U-test. **g-h**, Representative H&E staining (**g**) and quantification of BrdU-positive cells (**h**) from *VilCre^{ER};Apc$^{fl/fl}$;Kras$^{G12D/+}$* mice 3 days post-tamoxifen induction and subsequent daily treatment with vehicle (pink) or ZD6474 (aqua). Scale bar, 100 μm. n=4 mice per group. Data are ± s.e.m; P=0.20. One-tail Mann–Whitney U-test for **g**.

Supplementary Table 1

site	morphology	surg_resect	recurrencecat
colon	adenocarcinoma	no	no
colon	adenocarcinoma	yes	no
colon	adenocarcinoma	no	no
colon	adenocarcinoma	yes	no
colon	adenocarcinoma	no	no
rectum/rectosigmoid	adenocarcinoma	yes	no
rectum/rectosigmoid	adenocarcinoma	no	no
rectum/rectosigmoid	adenocarcinoma	yes	no
colon	adenocarcinoma	yes	no
rectum/rectosigmoid	adenocarcinoma	yes	no
rectum/rectosigmoid	adenocarcinoma	yes	no
colon	adenocarcinoma	yes	no
rectum/rectosigmoid	adenocarcinoma	yes	no
colon	adenocarcinoma	yes	no
colon	adenocarcinoma	yes	no
rectum/rectosigmoid	adenocarcinoma	yes	no
rectum	adenocarcinoma	yes	no
colon	adenocarcinoma	yes	yes
colon	adenocarcinoma	no	yes
colon	adenocarcinoma	yes	yes
colon	adenocarcinoma	yes	yes
colon	adenocarcinoma	yes	yes
rectum/rectosigmoid	adenocarcinoma	yes	yes
colon	adenocarcinoma	no	yes
rectum/rectosigmoid	adenocarcinoma	yes	yes
colon	adenocarcinoma	no	yes
rectum/rectosigmoid	adenocarcinoma	yes	yes

Supplementary Table 2

	High (N=42)	Low (N=240)	Total (N=282)	p value
**Age**				0.109
Mean (SD)	66.524 (11.847)	69.529 (11.053)	69.082 (11.204)	
Range	39.000 - 87.000	37.000 - 93.000	37.000 - 93.000	
**Sex**				0.160
F	24 (57.1%)	109 (45.4%)	133 (47.2%)	
M	18 (42.9%)	131 (54.6%)	149 (52.8%)	
**Tumor.differentiation**				0.472
1	1 (2.4%)	15 (6.2%)	16 (5.7%)	
2	39 (92.9%)	207 (86.2%)	246 (87.2%)	
3	2 (4.8%)	18 (7.5%)	20 (7.1%)	
**Lymphovascular.invasion**				0.170
N	35 (83.3%)	217 (90.4%)	252 (89.4%)	
Y	7 (16.7%)	23 (9.6%)	30 (10.6%)	
**Perineural.invasion**				0.345
N	42 (100.0%)	235 (97.9%)	277 (98.2%)	
Y	0 (0.0%)	5 (2.1%)	5 (1.8%)	
**Location**				0.044
Left	7 (16.7%)	32 (13.3%)	39 (13.8%)	
Rectosigmoid	7 (16.7%)	11 (4.6%)	18 (6.4%)	
Rectum	0 (0.0%)	1 (0.4%)	1 (0.4%)	
Right	14 (33.3%)	105 (43.8%)	119 (42.2%)	
Sigmoid	12 (28.6%)	62 (25.8%)	74 (26.2%)	
Transverse	2 (4.8%)	29 (12.1%)	31 (11.0%)	
**LNs.examined**				0.154
Mean (SD)	14.881 (7.781)	16.942 (8.762)	16.635 (8.641)	
Range	3.000 - 39.000	2.000 - 49.000	2.000 - 49.000	
**Rec.Status**				0.005
No	31 (73.8%)	215 (89.6%)	246 (87.2%)	
Yes	11 (26.2%)	25 (10.4%)	36 (12.8%)	
**RFS**				0.317
Mean (SD)	5.842 (4.549)	6.573 (4.327)	6.464 (4.360)	
Range	0.240 - 17.380	0.250 - 20.570	0.240 - 20.570	
**MMR**				0.069
MMR-d	4 (9.5%)	52 (21.7%)	56 (19.9%)	
MMR-p	38 (90.5%)	188 (78.3%)	226 (80.1%)	
