# Supplementary material for: Epithelial TGFβ engages growth-factor signalling to circumvent apoptosis and drive intestinal tumourigenesis with aggressive features
Source: Nat Commun. 2022 Dec 7;13:7551. doi: 10.1038/s41467-022-35134-3 (PMC9729215; doi:10.1038/s41467-022-35134-3)
Supplement: Supplementary file 3 — Reporting Summary [file 41467_2022_35134_MOESM3_ESM.pdf]

nature portfolio

Corresponding author(s): Owen Sansom

Last updated by author(s): Oct 15, 2022

Reporting Summary

Nature Portfolio wishes to improve the reproducibility of the work that we publish. This form provides structure for consistency and transparency in reporting. For further information on Nature Portfolio policies, see our Editorial Policies and the Editorial Policy Checklist.

Statistics

For all statistical analyses, confirm that the following items are present in the figure legend, table legend, main text, or Methods section.

n/a	Confirmed	
☐	☒	The exact sample size (*n*) for each experimental group/condition, given as a discrete number and unit of measurement
☐	☒	A statement on whether measurements were taken from distinct samples or whether the same sample was measured repeatedly
☐	☒	The statistical test(s) used AND whether they are one- or two-sided<br>*Only common tests should be described solely by name; describe more complex techniques in the Methods section.*
☒	☐	A description of all covariates tested
☐	☒	A description of any assumptions or corrections, such as tests of normality and adjustment for multiple comparisons
☐	☒	A full description of the statistical parameters including central tendency (e.g. means) or other basic estimates (e.g. regression coefficient) AND variation (e.g. standard deviation) or associated estimates of uncertainty (e.g. confidence intervals)
☐	☒	For null hypothesis testing, the test statistic (e.g. *F*, *t*, *r*) with confidence intervals, effect sizes, degrees of freedom and *P* value noted<br>*Give P values as exact values whenever suitable.*
☒	☐	For Bayesian analysis, information on the choice of priors and Markov chain Monte Carlo settings
☒	☐	For hierarchical and complex designs, identification of the appropriate level for tests and full reporting of outcomes
☐	☒	Estimates of effect sizes (e.g. Cohen's *d*, Pearson's *r*), indicating how they were calculated

Our web collection on statistics for biologists contains articles on many of the points above.

Software and code

Policy information about availability of computer code

Data collection	qPCR was analysed using CFX Maestro Software 2.3. Power analyses were carried out using G* power software package 3.1.9.4 (HHU Dusseldorf).
Data analysis	Data analysis was undertaken using Graphpad Prism version 8. Normalization and differential analysis of RNA-seq data was carried out using DESeq2 (v1.24.0) package. Microarray data were normalised using Robust Multi-array Average (RMA) in Partek Genomics Suite software (v6.6; Partek Inc.) and differential analysis was performed using Limma (v3.40.6) package. The codes and scripts are available on https://github.com/MolecularPathologyLab/T1-Alk5ca. The custom code is written in R 3.6.3. For image analysis Image J, version 1.52h and Perkin Elmer Harmony high-content and imaging analysis software, version 4.8 were used. Digital image analysis of whole slide images was conducted in QuPath 1.2.0.

For manuscripts utilizing custom algorithms or software that are central to the research but not yet described in published literature, software must be made available to editors and reviewers. We strongly encourage code deposition in a community repository (e.g. GitHub). See the Nature Portfolio guidelines for submitting code & software for further information.

Data

Policy information about availability of data

All manuscripts must include a data availability statement. This statement should provide the following information, where applicable:

- Accession codes, unique identifiers, or web links for publicly available datasets
- A description of any restrictions on data availability
- For clinical datasets or third party data, please ensure that the statement adheres to our policy

Raw and processed RNA sequencing data that support the findings in this study and available on GEO under accession number GSE182019 [https://

www.ncbi.nlm.nih.gov/geo/query/acc.cgi?acc=GSE182019]. Large raw image/micrograph files that support the findings in this study are not provided with the source data and are available from the corresponding authors upon request. pT1 clinicopathological information are provided in Supplementary Table1, and raw and processed transcriptional data is available on GEO under accession number GSE162667 [https://www.ncbi.nlm.nih.gov/geo/query/acc.cgi?acc=GSE162667]. Raw and processed transcriptional data from FOCUS cohort data are available on GEO under accession number GSE156915 [https://www.ncbi.nlm.nih.gov/geo/query/acc.cgi?acc=GSE156915]. The CRC cohort was comprised of 282 stage II patients and was previously described in Oncotarget. 2020 Feb 25; 11(8): 813–824; doi: 10.18632/oncotarget.27491. Source data are provided with this paper.

Field-specific reporting

Please select the one below that is the best fit for your research. If you are not sure, read the appropriate sections before making your selection.

- [x] Life sciences
- [ ] Behavioural & social sciences
- [ ] Ecological, evolutionary & environmental sciences

For a reference copy of the document with all sections, see nature.com/documents/nr-reporting-summary-flat.pdf

Life sciences study design

All studies must disclose on these points even when the disclosure is negative.

Sample size	For all in vivo experiments, power analyses were carried out to determine cohort sizes based upon effect size and SD derived from unpublished experiments in similar GA models previously carried out within the lab, and from early pilot studies which were carried out within experimental and control cohorts. Power analyses were carried out using the G* power software package 3.1.9.4 (HHU Dusseldorf), typically defining alpha=0.05 and beta=0.2. For animal studies this also respected the limited use of animals in line with the 3R system: Replacement, Reduction, Refinement.
Data exclusions	No data were excluded, unless mentioned otherwise.
Replication	For all organoid-related experiments, treatments were repeated twice on a minimum of 3 separate batches of organoids (n=3 mice) to verify reproducibility of the experimental findings. Tissue sections from 4 individual animals per genotype (n=4) were stained for all immunohistochemistry, immunofluorescence and in-situ hybridisation. All other experiments were performed on biological replicates of >3 mice to confirm reproducibility of experimental findings.
Randomization	To minimise genetic variability, all experimental and control animals were either generated on a pure, inbred genetic background, or where that was not possible, were generated from individual breeding colonies. Control and experimental animals were co-housed independent of genotype and cohorts were comprised of a balance of both male and female animals. In order to reduce the impact of covariates such as gender or housing, animals were recruited to treatment groups in a partially randomised manner while taking these factors into account. For all organoid experiments, organoid cultures of the same genetic background were treated in parallel ie. Apc-mutant line #1 + vehicle, Apc-mutant line #1 + drug.
Blinding	For animal welfare reasons, researchers were not blinded to genotype during study and data collection. The investigator(s) were blinded to genotype or treatment during data analysis. For organoid experiments, researchers were blinded to genotype during study and data collection, but not blinded to treatment or genotype during data analysis.

Reporting for specific materials, systems and methods

We require information from authors about some types of materials, experimental systems and methods used in many studies. Here, indicate whether each material, system or method listed is relevant to your study. If you are not sure if a list item applies to your research, read the appropriate section before selecting a response.

Materials & experimental systems

n/a	Involved in the study	
	x	Antibodies
x		Eukaryotic cell lines
x		Palaeontology and archaeology
	x	Animals and other organisms
	x	Human research participants
x		Clinical data
x		Dual use research of concern

Methods

n/a	Involved in the study	
x		ChIP-seq
x		Flow cytometry
x		MRI-based neuroimaging

Antibodies

Antibodies used	Antibody, catalog number and dilutions used for immunohistochemistry included in the manuscript.<br>β-catenin (1:100; BD Biosciences, #610154)<br>Lysozyme (1:500; Agilent, #A0099)<br>Ly6G (1:60,000, BioxCell, #BE0075-1)

	p-Smad3 (1:50, Abcam, #ab52903)<br>BrdU (1:250, BD Biosciences, #347580)<br>CD3 (1:100, Abcam, #ab16669)<br>Cleaved Caspase3 (Asp175) (1:300; CST, #9661)<br>F4/80 (1:100, Abcam, #ab6640)<br>CD31 (1:75, Abcam, #ab28364)<br>p-Erk (1:400; Cell Signaling Technology, #9101)
Validation	Antibody, catalog number and dilutions used for immunohistochemistry/immunofluorescence included in the manuscript.<br>1. β-catenin (1:100; BD Biosciences, #610154); validated by WB, IHC, IP, IF. Species Reactivity: Human, Mouse, Rat, Dog, Chicken.<br>2. Lysozyme (1:500; Dako, #A0099); validated by IHC. Species Reactivity: Human, Mouse.<br>3. p-Smad3 (1:50, Abcam, #ab52903); validated by IHC, WB and IF. Species Reactivity: Human, Mouse.<br>4. BrdU (1:250, BD Biosciences, #347580); validated by IHC, Flow Cytometry. Species Reactivity: Species independent.<br>5. CD3 (1:100, Abcam, #ab16669); validated by IHC, WB, Flow Cytometry and IF. Species Reactivity: Human, Mouse, Rat.<br>6. F4/80 (1:100, Abcam, #ab6640); validated by Flow Cytometry, IF. Species Reactivity: Mouse.<br>7. Cleaved Caspase3 (Asp175) (1:300; CST, #9661); Validated by WB, Flow Cytometry, IP, IHC and IF. Species Reactivity: Human, Mouse, Rat, Monkey.<br>8. CD31 (1:75, Abcam, #ab28364); validated by IHC. Species Reactivity: Mouse, Human and Pig.<br>9. p-Erk (1:400; Cell Signaling Technology, #9101); validated by IHC, WB, IF. Species reactivity: Human, Mouse, Rat Hamster, Monkey Mink, Drosophila melanogaster, Zebrafish, Bovine, Pig and C. elegans.<br>10. Ly6G (1:60,000, BioxCell, #BE0075-1); validated by IHC, IF and Flow cytometry. Species reactivity: Species independent.

Animals and other organisms

Policy information about studies involving animals; ARRIVE guidelines recommended for reporting animal research

Laboratory animals	Mice of both sexes from 2-6 months old C57/BL6J mice were used.
Wild animals	No wild animals were used in the study
Field-collected samples	No field-collected samples were used in this study.
Ethics oversight	All animal experiments were performed in accordance with UK Home Office regulations (under project licence 70/8646) and adherence to the ARRIVE guidelines and were subject to review by the Animal Welfare and Ethical Review Board of the University of Glasgow.

Note that full information on the approval of the study protocol must also be provided in the manuscript.

Human research participants

Policy information about studies involving human research participants

Population characteristics	Colon tumours were collected from patients diagnosed with T1 CRC who underwent radical surgery.
Recruitment	The pT1 samples were collected via the Northern Ireland Biobank (study reference: NIB16-0215), which has approval from ORECNI (16/NI/0030) to collect, store and distribute samples to researchers
Ethics oversight	Approval was given from ORECNI (16/NI/0030) to collect, store and distribute samples to researchers. Written informed consent from patients was obtained prior to acquisition of the tissue sample for research use.

Note that full information on the approval of the study protocol must also be provided in the manuscript.
